# Impact of population size on early adaptation in rugged fitness landscapes

**DOI:** 10.1098/rstb.2022.0045

**Published:** 2023-05-22

**Authors:** Richard Servajean, Anne-Florence Bitbol

**Affiliations:** ^1^ Institute of Bioengineering, School of Life Sciences, École Polytechnique Fédérale de Lausanne (EPFL), 1015 Lausanne, Switzerland; ^2^ SIB Swiss Institute of Bioinformatics, 1015 Lausanne, Switzerland

**Keywords:** fitness landscapes, epistasis, stochastic simulations, mathematical modelling, adaptation, finite-size effects

## Abstract

Owing to stochastic fluctuations arising from finite population size, known as genetic drift, the ability of a population to explore a rugged fitness landscape depends on its size. In the weak mutation regime, while the mean steady-state fitness increases with population size, we find that the height of the first fitness peak encountered when starting from a random genotype displays various behaviours versus population size, even among small and simple rugged landscapes. We show that the accessibility of the different fitness peaks is key to determining whether this height overall increases or decreases with population size. Furthermore, there is often a finite population size that maximizes the height of the first fitness peak encountered when starting from a random genotype. This holds across various classes of model rugged landscapes with sparse peaks, and in some experimental and experimentally inspired ones. Thus, early adaptation in rugged fitness landscapes can be more efficient and predictable for relatively small population sizes than in the large-size limit.

This article is part of the theme issue ‘Interdisciplinary approaches to predicting evolutionary biology’.

## 1. Introduction

Natural selection drives populations towards higher fitness (i.e. reproductive success), but actual fitness landscapes (representing fitness versus genotype [1,2]) can possess several distinct local maxima or peaks. Such rugged fitness landscapes arise from epistasis, i.e. interactions between genetic variants [3–6], especially from reciprocal sign epistasis [4], where two mutations together yield a benefit while they are deleterious separately, giving rise to a fitness valley [7,8]. While the high dimension of genotype space makes it challenging to probe fitness landscapes [9,10], experimental evidence has been accumulating for frequent landscape ruggedness [6–8,10–15]. This strongly impacts the predictability of evolution [5,6,14]. Populations can remain stuck at a local fitness peak, thus preventing further adaptation. Which local peak is reached depends on the starting point, on the mutations that occurred, on their order, and on whether they took over or not. Historical contingency may thus play important roles.

In a constant environment, if mutations are rare, the evolution of a homogeneous population of asexual microorganisms can be viewed as a biased random walk in genotype space, and thus on the associated fitness landscape [16]. Indeed, random mutations can either fix (i.e. take over) or get extinct, depending on how mutant fitness compares to wild-type fitness (natural selection) and on stochastic fluctuations owing to finite population size (genetic drift). In the weak mutation regime, mutations are rare enough for their fate to be sealed before a new mutation takes place. Thus, the population almost always has a single genotype, i.e. it is monomorphic. When a mutant fixes, it becomes the new wild-type: the population has moved in genotype space—hence the biased random walk in genotype space. If in addition natural selection is strong [16,17], only beneficial mutations, which increase fitness, can fix. In this regime, the random walk describing the evolution of the population can only go upwards in fitness. Such *adaptive walks* (AWs) [16] have been extensively studied [18]. Strong selection neglects the possibility that deleterious or neutral mutations may fix owing to genetic drift, which is appropriate only for very large populations [19]. Conversely, if the strong selection hypothesis is dropped, deleterious mutations may fix [20,21], and a population’s ability to explore its fitness landscape depends on its size, which determines the amplitude of genetic drift [22]. How does the interplay between genetic drift and natural selection [23] impact adaptation of a finite-size population on rugged fitness landscapes? In particular, is adaptation always more efficient for larger populations?

To address this question, we consider homogeneous populations of constant size *N*, which evolve in the weak mutation regime, either through the Moran model [22,24], or through the Wright–Fisher model under the diffusion approximation [25,26]. The steady-state properties of such evolution have been studied, in particular the stationary distribution of states [27,28] and their dynamical neighbourhoods [29]. The mean steady-state fitness monotonically increases with population size (see the electronic supplementary material, S1 and figure S1), so the long-term outcome of evolution becomes more optimal and predictable when population size increases. A very large finite population will reach the highest fitness maximum of the landscape, but this may take very long, owing to the difficulty of crossing fitness valleys for large populations with rare mutations. Here, we investigate the dynamics of adaptation before steady state is reached, and we ask how population size impacts early adaptation.

We focus on early adaptation, by considering the first fitness peak encountered starting from a randomly chosen genotype. We mainly study the fitness of this first encountered peak, and we also discuss the time needed to reach it. Both have been extensively studied for AWs [18]. We find that, in contrast to the steady-state fitness, the fitness $\bar{h}$ of the first encountered peak, averaged over starting genotypes, does not always increase with population size *N*. Thus, adaptation is not always more efficient for larger populations. Furthermore, we observe a wide variety of behaviours of $\bar{h}$ with *N*, even among small and simple rugged landscapes. We show that the accessibility of the different fitness peaks is a key ingredient to determine whether $\bar{h}$ is larger or smaller for large *N* than for *N* = 1. We find that the ensemble mean $\left\langle \bar{h}\right\rangle$ of $\bar{h}$ over different model landscapes often features a maximum for a finite value of *N*, showing that early adaptation is often most efficient for intermediate *N*. This effect occurs in rugged landscapes with low densities of peaks, is particularly important for large genomes with pairwise epistasis, and matters for larger populations when genomes are large. More generally, such finite-size effects extend to larger populations when many mutations are (almost) neutral. These situations are relevant in practice. Furthermore, our main conclusions hold for multiple experimental and experimentally motivated landscapes.

## 2. Methods

### (a) Model

We consider a homogeneous population comprising a constant number *N* of asexual haploid individuals, e.g. bacteria. We assume that their environment is constant, and we neglect interactions between genotypes (individual types, characterized by the state of all genes) and frequency-dependent selection. Each genotype is mapped to a fitness through a fitness landscape [1,2], which is static under these hypotheses [6]. We consider various rugged fitness landscapes (see Results).

Evolution is driven by random mutations, corresponding to a genotype change in one organism. The genotype of each organism is described by a sequence of *L* binary variables, taking values 0 or 1, which correspond to nucleotides, amino acids, genes or any other relevant genetic unit. The binary state is a simplification [30], which can represent the most frequent state (0) and any variant (1). Genotype space is then a hypercube with 2^*L*^ nodes, each of them having *L* neighbours accessible by a single mutation (i.e. a substitution from 0 to 1 or vice-versa at one site). Note that we do not model insertions or deletions. For simplicity, we further assume that all substitutions have the same probability.

Because the population size *N* is finite and there is no frequency-dependent selection, each mutant lineage either fixes (i.e. takes over the population) or gets extinct, excluding coexistence between quasi-stable clades [31]. We focus on the weak mutation regime, defined by *Nμ* ≪ 1 where *μ* denotes mutation probability per site and per generation. Mutations are then rare enough for their fate to be sealed before any new mutation takes place. Thus, the population almost always has a single genotype, i.e. it is monomorphic, excluding phenomena such as clonal interference [31–33]. When a mutant fixes, it becomes the new wild-type. In this framework, the evolution of the population by random mutations, natural selection and genetic drift can be viewed as a biased random walk in genotype space [16]. A mutation followed by fixation is one step of this random walk, where the population hops from one node to another in genotype space. To describe the fixation of a mutation in a homogeneous population of size *N* under genetic drift and natural selection, we consider two population genetics models: the Moran model [22,24] and the Wright–Fisher model under the diffusion approximation [25,26], yielding two specific walks in genotype space. We use these models within the origin-fixation approach [21], where the mutation fixation rate is written as the mutation origination rate times the fixation probability. Note that we assume that fitness is positive, as it represents division rate, requiring minor modifications for some fitness landscape models (§3b).

#### (i) Moran walk

In the Moran process, at each step, an individual is picked to reproduce with a probability proportional to its fitness, and an individual is picked to die uniformly at random [22,24]. The fixation probability of the lineage of one mutant individual with genotype *j* and fitness *f*_*j*_ in a wild-type population with genotype *i* and fitness *f*_*i*_ reads [22]:

2.1 $$\begin{array}{rl} P_{ij} & =\frac{1-\;f_{i}/\;f_{\;j}}{1-{\left(\;f_{i}/\;f_{\;j}\right)}^{N}}=\frac{1-{\left(1+s_{ij}\right)}^{-1}}{1-{\left(1+s_{ij}\right)}^{-N}}\;\mathrm{if}\;s_{ij}\neq 0\\ \;\mathrm{and}\;P_{ij} & =\frac{1}{N}\;\;\;\;\;\;\;\;\;\;\;\;\mathrm{if}\;s_{ij}=0, \end{array}\}$$

where *s*_*ij*_ = *f*_*j*_/*f*_*i*_ − 1. In the Moran walk, all mutations (substitutions at each site) are equally likely, and when a mutation arises, it fixes with probability *P*_*ij*_. If it does, the population hops from node *i* to node *j* in genotype space. The Moran walk is a discrete Markov chain, where time is in number of mutation events, and it is irreducible, aperiodic and positive recurrent (and thus ergodic). Hence, it possesses a unique stationary distribution towards which it converges for any initial condition [34,35]. It is also reversible [28,29]. Note that evolution in the strong selection weak mutation regime (large-*N* limit of the present case) yields absorbing Markov chains, with different properties.

#### (ii) Wright–Fisher walk

The Wright–Fisher model assumes non-overlapping generations, where the next generation is drawn by binomial sampling [26]. Under the diffusion approximation valid for large populations (*N* ≫ 1) and mutations of small impact (|*s*_*ij*_|≪ 1) [25,26], the fixation probability of mutant *j* reads:

2.2 $$\begin{array}{cc} & P_{ij}=\frac{1-e^{-2s_{ij}}}{1-e^{-2Ns_{ij}}}\;\mathrm{if}\;s_{ij}\neq 0\\ \mathrm{and}\; & P_{ij}=\frac{1}{N}\;\;\;\;\mathrm{if}\;s_{ij}=0. \end{array}\}$$

We use this formula similarly as above to define the Wright–Fisher walk, which is also an irreducible, aperiodic, positive recurrent and reversible discrete Markov chain converging to a unique stationary distribution. Note that we use it for all *N* and fitness values, but that it rigorously holds only under the diffusion approximation, i.e. under assumptions of large population and weak selection. In fact, the complete Wright–Fisher model is irreversible for large selection, although this does not impact the steady-state distribution of populations on fitness landscapes [36]. By contrast, the Moran fixation probability is exact within the Moran process.

### (b) Quantifying early adaptation

To investigate early adaptation, and its dependence on population size, we mainly focus on the height *h* of the walk, which is the fitness of the first encountered peak [18]. It depends on the initial node *i*, and also on what happens at each step of the walk. We consider the average $\bar{h}$ of *h* over many walks and over all possible initial nodes, assumed to be equally likely: this quantity globally characterizes early adaptation in the fitness landscape. Starting from a random node is relevant, e.g. after an environmental change which made the wild-type no longer optimal, and allows us to characterize early adaptation over the whole fitness landscape. We also study the impact of restricting the set of starting points to those with high fitness (see also [16]), which is relevant for small to moderate environmental changes. To assess the variability of *h*, we also consider its standard deviation *σ*_*h*_. Note that by definition, the height *h* is directly the fitness value of a peak.

In addition to $\bar{h}$, we study the walk length $\bar{\ell }$, and its time $\bar{t}$, which are, respectively, the mean number of successful fixations and of mutation events (leading to fixation or not) before the first peak is reached, with similar methods as for $\bar{h}$ (see the electronic supplementary material, S2).

#### (i) First step analysis

To express $\bar{h}$, we consider the first hitting times of the different peaks (local fitness maxima) of the landscape [35]. Denoting by *M* the set of all nodes that are local maxima and by *T*_*j*_ the first hitting time of *j* ∈ *M*, we introduce the probability *P*_*i*_(*T*_*j*_ = min [*T*_*k*_, *k* ∈ *M*]) that a walk starting from node *i* hits *j* before any other peak. Discriminating over all possibilities for the first step of the walk (first step analysis, FSA) yields

2.3 $$P_{i}(T_{\;j}=\min[T_{k},k\in M])=\left\{ \begin{array}{cc} 1\; & \mathrm{if}\;i=j\;,\;\\ 0\; & \mathrm{if}\;i\in M\;\mathrm{and}\;i\neq j,\;\\ \sum_{l\in G_{i}}{\tilde{P}}_{il}\;P_{l}(T_{\;j}=\min[T_{k},k\in M])\; & \mathrm{otherwise},\; \end{array}\right.$$

where *G*_*i*_ is the set of neighbours of *i* (i.e. the *L* genotypes that differ from *i* by only one mutation), while ${\tilde{P}}_{il}=P_{il}/\sum_{q\in G_{i}}P_{iq}$, where *P*_*il*_ is the fixation probability of the mutation from *i* to *l*, given by equations (2.1) or (2.2). Thus, ${\tilde{P}}_{il}$ is the probability to hop from *i* to *l* at the first step of the walk. Solving this system of 2^*L*^ *n*_*M*_ equations, where *n*_*M*_ is the number of local maxima in the fitness landscape, yields all the first hitting probabilities. This allows us to compute

2.4 $$\bar{h}=\frac{1}{2^{L}}\sum_{i\in G}{\bar{h}}_{i},\;\mathrm{with}\;{\bar{h}}_{i}=\sum_{\;j\in M}f_{\;j}\;P_{i}(T_{\;j}=\min[T_{k},k\in M]),$$

where *G* is the ensemble of all the nodes of the landscape. Note that if the landscape has only two peaks *j* and *k*, it is sufficient to compute *P*_*i*_(*T*_*j*_ < *T*_*k*_) for all *i*, which can be expressed from the fundamental matrix of the irreducible, aperiodic, positive recurrent and reversible Markov chain corresponding to the Moran or Wright–Fisher walk [29,35]. These first hitting probabilities also allow us to compute the standard deviation *σ*_*h*_ of *h*.

In practice, we solve equation (2.3) numerically using the NumPy function linalg.solve. Note, however, that since the number of equations increases exponentially with *L* and linearly with *n*_*M*_, this is not feasible for very large landscapes.

#### (ii) Stochastic simulations

We also perform direct stochastic simulations of Moran and Wright–Fisher walks based on equations (2.1) or (2.2), using a Monte Carlo procedure. Note that we simulate the embedded version of these Markov chains, where the transition probabilities to all neighbours of the current node are normalized to sum to one, avoiding rejected moves. The only exception is when we study the time *t* of the walks, which requires including mutations that do not fix.

#### (iii) Averaging over multiple fitness landscapes

To characterize adaptation in an ensemble of landscapes, we consider the ensemble mean $\left\langle \bar{h}\right\rangle$ of $\bar{h}$ by sampling multiple landscapes from the ensemble, and taking the average of $\bar{h}$, either computed by FSA or estimated by simulations.

## 3. Results

### (a) Early adaptation on *LK* fitness landscapes

The *LK* model (originally called *NK* model) describes landscapes with tunable epistasis and ruggedness [37]. In this model, the fitness of genotype $\sigma =(\sigma_{1},\sigma_{2},\ldots ,\sigma_{L})\in {\left\{0,1\right\}}^{L}$ reads:

3.1 $$f\;(\sigma )=\sum_{i=1}^{L}f_{i}({\left\{\sigma_{\;j}\right\}}_{\;j\in \nu_{i}}),$$

where $f_{i}({\left\{\sigma_{\;j}\right\}}_{\;j\in \nu_{i}})$ denotes the fitness contribution associated with site *i*, and *ν*_*i*_ is the set of epistatic partners of *i*, plus *i* itself. Here *L* is genome length, i.e. the number of binary units (genes or nucleotides or amino acids) that characterize genotype, while *K* is the number of epistatic partners of each site *i*—thus, for each *i*, *ν*_*i*_ comprises *K* + 1 elements. Unless mentioned otherwise, we consider *LK* landscapes where sets of partners are chosen uniformly at random, i.e. in a ‘random neighbourhood’ scheme [18], and each fitness contribution $f_{i}({\left\{\sigma_{\;j}\right\}}_{\;j\in \nu_{i}})$ is independently drawn from a uniform distribution between 0 and 1. Epistasis increases with *K*. For *K* = 0, all sites contribute additively to fitness. For *K* = 1, each site *i* has one epistatic partner, whose state impacts *f*_*i*_. For *K* > 1, there is higher-order epistasis. For *K* = *L* − 1, all fitness contributions change when the state of one site changes, yielding a House of Cards landscape [38,39] where the fitnesses of different genotypes are uncorrelated.

How does finite population size *N* impact the average height $\bar{h}$ of the first peak reached by an adapting population starting from a uniformly chosen genotype? We first tackle this question in *LK* landscapes with *L* = 3 and *K* = 1, which are small and simple rugged landscapes.

#### (i) Average over *LK* landscapes with *L* = 3 and *K* = 1

**Figure 1.**
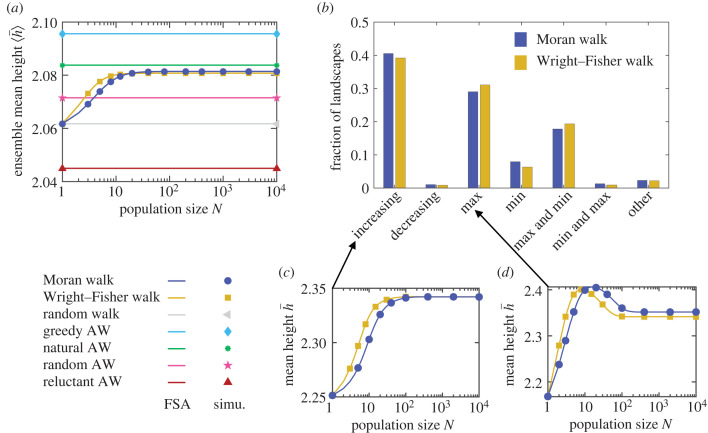
Impact of population size on early adaptation in *LK* landscapes with *L* = 3 and *K* = 1. (*a*) Ensemble mean height $\left\langle \bar{h}\right\rangle$ of the first fitness peak reached when starting from a uniformly chosen initial node versus population size *N* for various walks. Lines: numerical resolutions of the FSA equations for each landscape; markers: simulation results averaged over 100 walks per starting node in each landscape. In both cases, the ensemble average is performed over 5.6 × 10^5^ landscapes. (*b*) Distribution of behaviours displayed by $\bar{h}$ versus *N* for the Moran and Wright–Fisher walks over the 9.3 × 10^4^ landscapes with more than one peak from an ensemble of 2 × 10^5^ landscapes. Classes of behaviours of $\bar{h}$ versus *N* are: monotonically increasing or decreasing, one maximum, one minimum, one maximum followed by a minimum at larger *N* (max and min), vice-versa (min and max), and more than two extrema (other). In each landscape, we numerically solve the FSA equations for various *N*. (*c*,*d*) $\bar{h}$ versus *N* is shown in two example landscapes (see the electronic supplementary material, table S1), landscape A yielding a monotonically increasing behaviour (*c*) and landscape B yielding a maximum (*d*). Same symbols as in (*a*); simulation results averaged over 10^5^ (*c*) and 5 × 10^5^ (*d*) walks per starting node. (Online version in colour.)

Figure 1*a* shows that the ensemble mean $\left\langle \bar{h}\right\rangle$ of $\bar{h}$ over these landscapes monotonically increases with *N* both for the Moran and for the Wright–Fisher walk. FSA and stochastic simulation results (see Methods) are in very good agreement. Thus, on average over these landscapes, larger population sizes make early adaptation more efficient. This is intuitive because natural selection becomes more and more important compared to genetic drift as *N* increases, biasing the walks toward larger fitness increases. Figure 1*a* also shows $\left\langle \bar{h}\right\rangle$ for the various AWs [18] defined in the electronic supplementary material, S3, and for the pure random walk. For *N* = 1, the Moran and Wright–Fisher walks reduce to pure random walks, since all mutations are accepted. For *N* → ∞, where the Moran and Wright–Fisher walks become AWs, $\left\langle \bar{h}\right\rangle$ is close to the value obtained for the natural AW, where the transition probability from *i* to *j* is proportional to *s*_*ij*_ = *f*_*j*_/*f*_*i*_ − 1 if *s*_*ij*_ > 0 and vanishes if *s*_*ij*_ < 0 [17,40,41]. Indeed, when *N* → ∞, the Moran (resp. Wright–Fisher) fixation probability in equation (2.1) (resp. equation (2.2)) converges to *s*_*ij*_/(1 + *s*_*ij*_) (resp. 1 − exp (− 2*s*_*ij*_)) if *s*_*ij*_ > 0 and to 0 otherwise. If in addition 0 < *s*_*ij*_ ≪ 1 while *Ns*_*ij*_ ≫ 1, then they converge to *s*_*ij*_ and 2*s*_*ij*_, respectively, and both become equivalent to the natural AW. The slight discrepancy between the asymptotic behaviour of the Moran and Wright–Fisher walks and the natural AW comes from the fact that not all *s*_*ij*_ satisfy |*s*_*ij*_|≪ 1 in these landscapes. Convergence to the large-*N* limit occurs when *Ns*_*ij*_ ≫ 1 for all relevant *s*_*ij*_, meaning that landscapes with near-neutral mutations will feature finite-size effects up to larger *N*. Besides, this convergence occurs for slightly larger *N* for the Moran walk than for the Wright–Fisher walk (see figure 1*a*). Indeed, if *s*_*ij*_ > 0, ln(1 + *s*_*ij*_) < 2*s*_*ij*_, so equation (2.2) converges to its large-*N* limit faster than equation (2.1), while if −0.79 < *s*_*ij*_ < 0, ln(1 + *s*_*ij*_) > 2*s*_*ij*_, so equation (2.2) tends to 0 faster than equation (2.1) for large *N* (*s*_*ij*_ < −0.79 is very rare and yields tiny fixation probabilities). Note that in figure 1*a*, the range of variation of $\left\langle \bar{h}\right\rangle$ with *N* is small, but this is landscape-dependent (see figure 3).

How does the time needed to reach the first peak depend on *N* in *LK* landscapes with *L* = 3 and *K* = 1? First, the ensemble mean length $\left\langle \bar{\ell }\right\rangle$, defined as the mean number of mutation fixations before the first peak is reached, decreases before saturating as *N* increases (see the electronic supplementary material, figure S2a). Indeed, when *N* increases, the walk becomes more and more biased towards increasing fitness. Conversely, the ensemble mean time $\left\langle \bar{t}\right\rangle$, defined as the mean number of mutation events (fixing or not) before the first peak is reached, increases with *N* (see the electronic supplementary material, figure S2b). Indeed, many mutations are rejected for large *N*. Moreover, at a given *s*_*ij*_, fixation probabilities (equations (2.1) and (2.2)) decrease as *N* increases. Note that for *Ns*_*ij*_ ≫ 1 and *s*_*ij*_ ≪ 1, the limit of equation (2.1) is *s*_*ij*_ while that of equation (2.2) is 2*s*_*ij*_, explaining why the large-*N* limit of $\left\langle \bar{t}\right\rangle$ is about twice as large for the Moran than for the Wright–Fisher walk. Note that, more generally, a factor of 2 differs between the diffusion limits of the fixation probabilities of the Moran and the Wright–Fisher models. It arises from a difference in the variance in offspring number [22]. Finally, since mutations occur proportionally to *N*, the actual time needed by the population to reach the first peak is proportional to $\langle \bar{t}\rangle /N$, which decreases with *N* (see the electronic supplementary material, figure S2c).

#### (ii) Diversity among *LK* landscapes with *L* = 3 and *K* = 1

How much does the population-size dependence of $\bar{h}$ depend on the specific landscape considered? To address this question, we focus on landscapes that have more than one peak (46% of *L* = 3, *K* = 1 landscapes), since with a single peak, $\bar{h}$ is always equal to the fitness of that peak. Interestingly, figure 1*b* shows that $\bar{h}$ does not always monotonically increase with *N*. In fact, this expected case occurs only for about 40% of the landscapes with more than one peak (e.g. figure 1*c*), and $\bar{h}$ can exhibit various behaviours versus *N*. Around 30% of landscapes with more than one peak yield a single maximum of $\bar{h}$ versus *N* (e.g. figure 1*d*). For these landscapes, there is a specific finite value of *N* that optimizes early adaptation. While some landscapes yield multiple extrema of $\bar{h}$ versus *N*, the absolute amplitude of secondary extrema is generally negligible. Indeed, when $\bar{h}$ versus *N* displays two or more extrema, the mean ratio of the amplitude of the largest extremum to that of other extrema is larger than 20. Here, the amplitude of the *i*th extremum starting from *N* = 1, observed at *N* = *N*_*i*_, is computed as the mean of $A_{i}=|\bar{h}(N_{i})-\bar{h}(N_{i-1})|$ and *A*_*i*+1_ (where *N*_0_ = 1 and *N*_*i*+1_ → ∞ for the last extremum). Figure 1*b* shows that in 14% of landscapes with more than one peak, the Moran and the Wright–Fisher walks exhibit different behaviours. However, the scale of these differences is negligible. As illustrated by figure 1*b*, studying the behaviour of $\bar{h}$ versus *N* could be useful to characterize and classify fitness landscapes, and potentially complementary to epistasis measures in [10,42–45].

The mean length $\bar{\ell }$ and time $\bar{t}$ of the walk also vary across landscapes, but the same overall trends as for the ensemble mean length and time are observed (see the electronic supplementary material, figure S2d–i).

#### (iii) Impact of the starting set of genotypes

So far, we have considered the average $\bar{h}$ of *h* over all possible initial genotypes, assumed to be equally likely. What is the impact of restricting the set of possible starting points to those with high fitness? This question is relevant to adaptation after small to moderate sudden environmental changes [16], where the wild-type is no longer optimal, but still has relatively high fitness. To address it, we choose starting points uniformly among the *n* fittest genotypes. In the electronic supplementary material, figure S3, we study the same landscapes as in figure 1, varying *n* between 1 and 2^*L*^. For *n* = 2^*L*^, all genotypes can be starting points, as before. The behaviour of the ensemble mean $\left\langle \bar{h}\right\rangle$ versus *N* is similar across the different sets of starting points (electronic supplementary material, figure S3a). Electronic supplementary material, figure S3b further shows that $\bar{h}$ versus *N* always monotonically increases for the landscape of figure 1*c*. Finally, in the electronic supplementary material, figure S3c, $\bar{h}$ versus *N* displays an intermediate maximum for the landscape of figure 1*d*, except for *n* = 1 and *n* = 2. In these cases, the possible starting points are either only the absolute peak of the landscape, or itself and one of its neighbours that has a higher fitness than the small peak, so the latter rarely comes into play. This is also why the values of $\bar{h}$ are substantially larger for *n* = 1 and *n* = 2 than in other cases. Overall, these results suggest that our main conclusions are robust to varying the set of starting genotypes.

#### (iv) Predicting the overall behaviour of $\bar{h}$

Why do different *L* = 3, *K* = 1 landscapes yield such diverse behaviours of $\bar{h}$ versus *N*? To address this question, let us first focus on the *overall behaviour* of $\bar{h}$ versus *N*, i.e. on whether $\bar{h}$ is larger for *N* → ∞ (*overall increasing*) or for *N* = 1 (*overall decreasing*). This distinction is robust across the Moran and Wright–Fisher walks, as their overall behaviour differs only in 0.4% of the landscapes with more than one peak.

Let us focus on the landscapes with two peaks (99.5% of the landscapes with more than one peak) for simplicity: 87% of them yield an overall increasing behaviour of $\bar{h}$ versus *N*, as e.g. those featured in figure 1*c*,*d*. Intuitively, the higher a peak, the more attractive it becomes for large *N* given the larger beneficial mutations leading to it, and an overall increasing behaviour is thus expected. However, the opposite might happen if more paths with only beneficial mutations lead to the low peak than to the high peak—the low peak is then said to be *more accessible* than the high peak. Indeed, when *N* → ∞, only beneficial mutations can fix. Therefore, we compare the accessibility of the high peak and of the low peak.

**Figure 2.**
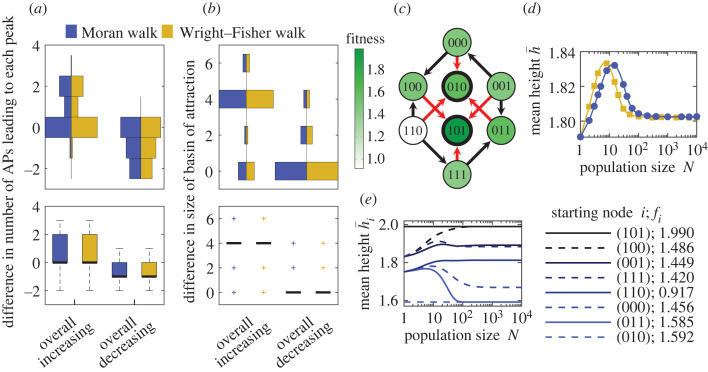
Accessibility of peaks and overall population-size dependence of early adaptation in *LK* landscapes with *L* = 3 and *K* = 1. All 9.2 × 10^4^ 2-peak landscapes from an ensemble of 2 × 10^5^ landscapes were sorted according to whether $\bar{h}$ versus *N* displays an overall increasing or decreasing behaviour (for the Moran and Wright–Fisher walks). (*a*,*b*) Distributions of two measures of differential accessibility of the high and low peaks (see main text) for these two classes of landscapes. Top panels: histograms (displayed vertically); bottom panels: associated box plots (bold black line: median; coloured boxes: 25th and 75th percentiles; dashed lines: minimum and maximum values that are not outliers; crosses: outliers). (*c*) Example landscape where both differential accessibility measures in (*a*,*b*) are 0. Bold circled nodes are peaks; arrows point towards fitter neighbours; red arrows point towards fittest neighbours. (*d*) $\bar{h}$ versus *N* for the Moran and Wright–Fisher walks in the landscape in (*c*). (*e*) Mean height $\bar{h_{i}}$ starting from each node *i* versus *N* for the Moran walk in the landscape in (*c*). Lines (*d*,*e*): numerical resolutions of FSA equations; markers (*d*): simulation results averaged over 10^5^ walks per starting node. (Online version in colour.)

In figure 2*a*,*b*, we show the distributions of two measures reflecting this differential accessibility in the two-peak landscapes with either overall increasing or overall decreasing dependence of $\bar{h}$ on *N*. The first measure (figure 2*a*) is the number of accessible paths (APs) [9] leading to the high peak minus the number of those leading to the low peak, where APs are paths comprising only beneficial mutations (note that APs included in other APs are not counted). The second measure (figure 2*b*) is the size of the basin of attraction of the high peak minus that of the low peak, where the basin of attraction is the set of nodes from which a greedy AW, where the fittest neighbour is chosen at each step, leads to the peak considered [46,47]. Figure 2 shows that landscapes displaying overall increasing behaviours tend to have a high peak more accessible than the low peak, and vice-versa for landscapes displaying overall decreasing behaviours. Quantitatively, 99% of the landscapes where both measures are positive or 0, but not both 0 (representing 75.2% of two-peak landscapes), yield an overall increasing behaviour. Moreover, 91% of the landscapes where both measures are negative or 0, but not both 0 (representing 5.7% of twopeak landscapes), yield an overall decreasing behaviour. Hence, differential accessibility is a good predictor of the overall behaviour of $\bar{h}$ versus *N*. Note that combining both measures is substantially more precise than using either of them separately (for instance, landscapes where the AP-based measure is strictly negative yield only 73% of overall decreasing behaviours).

However, for 15.2% of all two-peak landscapes, both differential accessibility measures are 0, and thus do not predict the overall behaviour. In practice, 70% of these tricky landscapes yield an overall increasing behaviour. One of those is shown in figure 2*c* and in a complementary representation in the electronic supplementary material, figure S4. It yields the $\bar{h}$ versus *N* curve in figure 2*d*. Note that another tricky, but rarer case, corresponds to landscapes where accessibility measures have strictly opposite signs (3.9% of two-peak landscapes).

#### (v) Finite-size effects on $\bar{h}$

Predicting the intermediate-*N* behaviour, e.g. the maximum in figure 2*d*, is more difficult than predicting the overall behaviour, and our accessibility measures do not suffice for this, nor do various ruggedness and epistasis measures from Szendro *et al.* [10], Aita *et al.* [42], Poelwijk *et al.* [43] and Ferretti *et al.* [44,45]. To understand this, let us consider the landscape in figure 2*c*. The mean heights ${\bar{h}}_{i}$ starting from each node *i* are displayed in figure 2*e*, showing that diverse behaviours combine to give that of $\bar{h}$. Starting from node (011), the only AP is to the low peak (010), so ${\bar{h}}_{i}$ decreases when *N* increases, but the quite small differences of fitnesses between (011) and its neighbours mean that relatively large values of *N* are required before this matters. Indeed, the convergence of fixation probabilities to their large-*N* limits occurs when *N*|*s*_*ij*_| ≫ 1 (see equations (2.1) and (2.2)). Conversely, starting from (100), the only AP is to the high peak (101), so ${\bar{h}}_{i}$ increases with *N*, starting at smaller values of *N* owing to the larger fitness differences involved, e.g. between (100) and (110). Such subtle behaviours, which depend on exact fitness values in addition to peak accessibility, yield the maximum in figure 2*d*.

For landscape B (figure 1*d*), which also yields a maximum of $\bar{h}$ at an intermediate value of *N*, the electronic supplementary material, figure S5 shows that the standard deviation *σ*_*h*_ of the height *h* reached from a uniformly chosen starting node features a minimum at a similar *N*, while the average $\bar{\sigma_{h_{i}}}$ over starting nodes *i* of the standard deviation of the height *h*_*i*_ starting from node *i* monotonically decreases when *N* increases. This corroborates the importance of the diversity of behaviours with starting nodes *i* in the finite-size effects observed. Moreover, the minimum in the standard deviation *σ*_*h*_ starting from any node means that early adaptation is more predictable for intermediate values of *N*. Note that *σ*_*h*_ and $\bar{\sigma_{h_{i}}}$ both decrease with *N* for landscape A, where $\bar{h}$ increases with *N* (figure 1*c*; electronic supplementary material, figure S5).

#### (vi) Magnitude of the overall variation of $\bar{h}$

We showed above that for two-peak fitness landscapes with *L* = 3 and *K* = 1, the differential accessibility of the peaks allows us to predict the overall behaviour of $\bar{h}$, i.e. the sign of $\Delta \bar{h}={\bar{h}}_{\infty }-\bar{h}(N=1)$, where ${\bar{h}}_{\infty }$ denotes the large-*N* limit of $\bar{h}$. What determines the magnitude of $\Delta \bar{h}$? The electronic supplementary material, figure S6 shows that it strongly correlates with the standard deviation $\sigma_{\;f_{M}}$ of the peak fitness values. This makes sense, as the range of $\bar{h}$ in a landscape is bounded by the fitness of the lowest peak and that of the highest peak.

#### (vii) Impact of *L* and *K*

So far we have focused on small *LK* landscapes with *L* = 3 and *K* = 1, which generally have one or two peaks. However, real fitness landscapes generally involve much larger genome lengths, *L* (number of binary units, representing genes, nucleotides or amino acids) and may involve larger numbers of epistatic partners, *K* and be more rugged. How do these two parameters impact $\left\langle \bar{h}\right\rangle$? First, $\left\langle \bar{h}\right\rangle$ increases linearly with *L* at *K* = 1, because all fitness values increase linearly with *L* in *LK* landscapes (equation (3.1); electronic supplementary material, figure S7a). For AWs, such a linear behaviour was analytically predicted with block neighbourhoods, and holds more generally when *L* ≫ *K* [18]. In addition, the electronic supplementary material, figure S7b shows that, at *L* = 20, $\left\langle \bar{h}\right\rangle$ features a maximum for an intermediate *K*, which depends on *N*. A similar maximum was observed in [18] for AWs.

**Figure 3.**
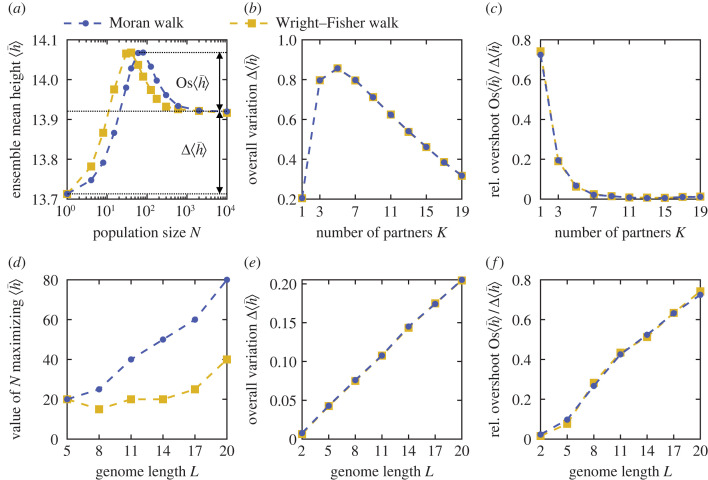
Impact of *L* and *K* on early adaption in *LK* landscapes. (*a*) Ensemble mean height $\left\langle \bar{h}\right\rangle$ of the first fitness peak reached when starting from a uniformly chosen initial node for *LK* landscapes with *L* = 20 and *K* = 1 versus population size *N* for the Moran and Wright–Fisher walks. The overall variation $\Delta \langle \bar{h}\rangle ={\left\langle \bar{h}\right\rangle }_{\infty }-\langle \bar{h}\rangle (N=1)$ and the overshoot $\mathrm{Os}\langle \bar{h}\rangle =\max\langle \bar{h}\rangle -{\left\langle \bar{h}\right\rangle }_{\infty }$ are indicated. (*b*) Overall variation $\Delta \langle \bar{h}\rangle$ versus the number of partners *K*, for *L* = 20. (*c*) Relative overshoot $\mathrm{Os}\langle \bar{h}\rangle /\Delta \langle \bar{h}\rangle$ versus *K*, for *L* = 20. (*d*) Value of the population size *N* that maximizes $\left\langle \bar{h}\right\rangle$ versus genome length *L* (i.e. number of binary loci), for *K* = 1. (*e*) Overall variation of $\left\langle \bar{h}\right\rangle$ (as in (*b*)), versus *L*, for *K* = 1. (*f*) Relative overshoot of $\left\langle \bar{h}\right\rangle$ (as in (*c*)), versus *L*, for *K* = 1. Markers connected by dashed lines are simulation results from 5 × 10^5^ walks (10^7^ for *L* = 2), each in a different landscape, generated along the way to save memory. The large-*N* limit ${\left\langle \bar{h}\right\rangle }_{\infty }$ is evaluated for *N* = 10^4^. (Online version in colour.)

While $\left\langle \bar{h}\right\rangle$ monotonically increases with *N* for *L* = 3 and *K* = 1 (figure 1*a*), a pronounced maximum appears at finite *N* for larger *L*, see figure 3*a*. To quantify how $\left\langle \bar{h}\right\rangle$ changes with *N*, we consider the overall variation $\Delta \langle \bar{h}\rangle ={\left\langle \bar{h}\right\rangle }_{\infty }-\langle \bar{h}\rangle (N=1)$ of $\left\langle \bar{h}\right\rangle$ between *N* = 1 and the large-*N* limit, as well as the overshoot of the large-*N* limit, $\mathrm{Os}\langle \bar{h}\rangle =\max\langle \bar{h}\rangle -{\left\langle \bar{h}\right\rangle }_{\infty }$ (see figure 3*a*). Their dependence on *K* and *L* is studied in figure 3. First, figure 3*b* shows that for *L* = 20, $\Delta \langle \bar{h}\rangle$ is maximal for *K* = 5, while figure 3*c* shows that the relative overshoot $\mathrm{Os}\langle \bar{h}\rangle /\Delta \langle \bar{h}\rangle$ is maximal for *K* = 1, and rapidly decreases for higher *K*. This is interesting, as *K* = 1 corresponds to pairwise interactions, highly relevant in protein sequences [48–50]. Next, we varied *L* systematically for *K* = 1 (see the electronic supplementary material, figure S8, for examples). A maximum of $\left\langle \bar{h}\right\rangle$ at finite *N* exists for *L* = 4 and above. The associated value of *N* increases with *L* (see figure 3*d*), but it remains modest for the values of *L* considered here. A key reason why finite-size effects matter for larger *N* when *L* increases is that more mutations are then effectively neutral, i.e. satisfy *N*|*s*_*ij*_| ≪ 1. This abundance of effectively neutral mutations is relevant in natural situations [51]. Furthermore, figure 3*e* shows that $\Delta \langle \bar{h}\rangle$ increases with *L*, and figure 3*f* shows that $\mathrm{Os}\langle \bar{h}\rangle /\Delta \langle \bar{h}\rangle$ also increases with *L*, exceeding 0.7 for *L* = 20. Thus, finite-size effects on early adaptation become more and more important as *L* is increased. This hints at important possible effects in real fitness landscapes, since genomes have many units (genes or nucleotides). As shown in the electronic supplementary material, figure S9, the density of peaks in the landscapes considered here decreases when *L* increases at *K* = 1, consistently with analytical results from Hwang *et al.* [52]. Therefore, maxima of $\bar{h}$ versus *N* are associated with rugged landscapes with sparse peaks.

Beyond these ensemble mean behaviours, we analyse how *L* and *K* impact the diversity of behaviours of $\bar{h}$ with *N* in the electronic supplementary material, figure S10. We find that the proportion of landscapes (with more than one peak) yielding a monotonically increasing $\bar{h}$ with *N* decreases steeply as *L* increases when *K* = 1, while the proportion of those yielding a maximum at intermediate *N* increases. An opposite, but less steep, trend is observed as *K* increases at *L* = 6. Thus, most landscapes yield a maximum of $\bar{h}$ at finite *N* when *L* is large enough and *K* = 1—the maximum of $\left\langle \bar{h}\right\rangle$ does not just arise from averaging over many landscapes. Besides, about 10% of landscapes with more than one peak yield an overall decreasing behaviour of $\bar{h}$ with *N* (i.e. ${\bar{h}}_{\infty }<\bar{h}(N=1)$) when *K* = 1 for all *L* > 2 considered. This behaviour becomes rarer when *K* increases at *L* = 6.

Finally, the impact of *L* and *K* on $\left\langle \bar{\ell }\right\rangle$ is shown in the electronic supplementary material, figure S7c,d: $\left\langle \bar{\ell }\right\rangle$ increases with *L* for *K* = 1, more strongly if *N* is small, and $\left\langle \bar{\ell }\right\rangle$ decreases as *K* increases at *L* = 20. Indeed, a larger *K* at constant *L* entails more numerous peaks and a larger *L* at constant *K* yields smaller peak density (the number of peaks increases less fast with *L* than the number of nodes). In addition, smaller *N* means more wandering in the landscapes and larger $\left\langle \bar{\ell }\right\rangle$. Note that for AWs in *LK* landscapes, a linear behaviour of $\left\langle \bar{\ell }\right\rangle$ versus *L* was analytically predicted with block neighbourhoods, and holds more generally for *L* ≫ *K* [18].

#### (viii) Impact of the neighbourhood scheme

So far, we have considered the random neighbourhood scheme [18] where epistatic partners are chosen uniformly at random. We find qualitatively similar behaviours in two other neighbourhood schemes (see electronic supplementary material, figure S11).

### (b) Extension to various model and experimental fitness landscapes

While the *LK* model is convenient as it allows us to explicitly tune epistasis and ruggedness, many other models exist, and natural fitness landscapes have been measured [15]. How general are our findings on the population-size dependence of early adaptation across fitness landscapes?

#### (i) Model fitness landscapes

We first consider different landscape models (see the electronic supplementary material, S4). In all of them, $\left\langle \bar{h}\right\rangle$ is overall increasing between *N* = 1 and the large-*N* limit, and either monotonically increases or features a maximum at intermediate *N*. This is consistent with our findings for *LK* landscapes, demonstrating their robustness. Specifically, we find maxima of $\left\langle \bar{h}\right\rangle$ for the *LKp* model, which includes neutral mutations [53], for the *LK* model with more than two states per site [30], and for the Ising model [44,54] (see the electronic supplementary material, figure S12a–c). Conversely, in models with stronger ruggedness (House of Cards landscapes, Rough Mount Fuji landscapes [10,55] with strong epistatic contributions and Eggbox landscapes [44]), we observe a monotonically increasing $\left\langle \bar{h}\right\rangle$ (see the electronic supplementary material, figure S12d–f). The electronic supplementary material, figure S13 shows that the density of peaks is generally smaller than 0.1 in the first three landscape ensembles and larger in the last three. This is consistent with our results for *LK* landscapes with *K* = 1 and different *L* (see above and the electronic supplementary material, figure S9), confirming that maxima of $\bar{h}$ versus *N* are associated with rugged landscapes with sparse peaks. Note that tuning the parameters of the model landscape ensembles considered here can yield various peak densities and behaviours, which we did not explore exhaustively.

#### (ii) Experimental and experimentally motivated landscapes

**Figure 4.**
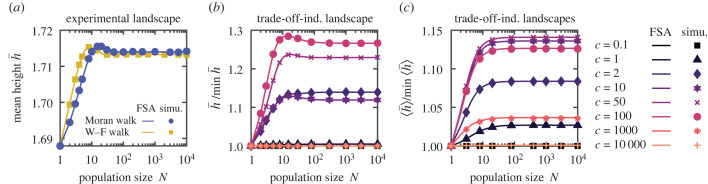
Impact of population size on early adaptation in an experimental landscape and in trade-off-induced landscapes. (*a*) Mean height $\bar{h}$ versus population size *N*, for the Moran and Wright–Fisher walks, in the experimental landscape from table S2 in [56]. In this study, the fitness landscape of *Escherichia coli* carrying dihydrofolate reductase from the malaria parasite *Plasmodium falciparum* was measured experimentally with or without the drug pyrimethamine. The focus was on four (wild-type or mutant) amino acids in dihydrofolate reductase that are important for pyrimethamine resistance, yielding a binary landscape with *L* = 4. The landscape studied here, without pyrimethamine, possesses two peaks. (*b*) Mean height $\bar{h}$ of the Moran walk, normalized by its minimal value, versus *N*, in a trade-off-induced landscape [57] with *L* = 6 (see the electronic supplementary material, table S2), for various (dimensionless) antibiotic concentrations *c*. (*c*) Ensemble mean height $\left\langle \bar{h}\right\rangle$ of the Moran walk, normalized by its minimal value, versus *N*, in an ensemble of tradeoff-induced landscapes [57] with *L* = 6 (see the electronic supplementary material, S4), for various *c*. Lines: numerical resolutions of FSA equations; markers: simulation data averaged over 10^5^ (*a*) and 10^4^ (*b*) walks per starting node. In (*c*), one walk is simulated per starting node in each landscape, and the ensemble average is over 1.5 × 10^6^ (resp. 5.6 × 10^5^) landscapes for simulations (resp. FSA) (10^5^ for *c* = 0.1, 10^3^ and 10^4^). (Online version in colour.)

We study $\bar{h}$ versus *N* in eight experimental rugged landscapes (see figure 4*a*; electronic supplementary material, figure S14). In all cases, we observe an overall increasing behaviour, most of them generally increasing, and two with a notable maximum at an intermediate size *N* (see figure 4*a*; electronic supplementary material, figure S14a). This is consistent with our results for model fitness landscapes, and shows their generality.

Trade-off-induced landscapes were introduced to model the impact of antibiotic resistance mutations in bacteria, in particular their tendency to increase fitness at high antibiotic concentration but decrease fitness without antibiotic [57,58] (see the electronic supplementary material, S4). These landscapes tend to be smooth at low and high antibiotic concentrations, but more rugged at intermediate ones, owing to the trade-off [57]. In a specific trade-off-induced landscape, we find that $\bar{h}$ versus *N* is flat for the smallest concentrations considered (the landscape has only one peak), becomes monotonically increasing for larger ones, and exhibits a maximum for even larger ones, before becoming flat again at very large concentrations (see figure 4*b*). In all cases, $\bar{h}$ versus *N* is overall increasing or flat. Besides, the ensemble average over a class of trade-off-induced landscapes (see the electronic supplementary material, S4) yields monotonically increasing behaviours of $\left\langle \bar{h}\right\rangle$ versus *N* for most concentrations, except the very small or large ones where it is flat (see figure 4*c*). The overall variation $\Delta \langle \bar{h}\rangle$ is largest (compared to the minimal value of $\left\langle \bar{h}\right\rangle$) for intermediate concentrations. These findings are consistent with our results for model and experimental fitness landscapes, further showing their generality.

## 4. Discussion

We studied early adaptation of finite populations in rugged fitness landscapes in the weak mutation regime, starting from a random genotype. We found that the mean fitness $\bar{h}$ of the first encountered peak depends on population size *N* in a non-trivial way, in contrast to the steady-state fitness which monotonically increases with *N*. We showed that the accessibility of different peaks plays a crucial part in whether $\bar{h}$ is larger in the large-*N* limit or for *N* = 1 in simple two-peaked landscapes. A key reason why $\bar{h}$ may not monotonically increase with *N* is that as *N* increases, Moran and Wright–Fisher walks lose possible paths as the fixation probability of deleterious mutations vanishes, while also becoming more biased towards larger fitness increases. These two conflicting effects of increasing *N* yield a trade-off. Accordingly, we observed that $\bar{h}$ versus *N* (and even the ensemble mean $\left\langle \bar{h}\right\rangle$) often features a maximum for intermediate *N*, especially in rugged fitness landscapes with small peak densities, where most nodes are relatively far from peaks. In these cases, early adaptation is more efficient, in the sense that higher peaks are found, for intermediate *N* than in the large-*N* limit. Studying the behaviour of $\bar{h}$ versus *N* could potentially be useful to characterize and classify landscapes.

Our results hold both for the Moran model, and for the Wright–Fisher model in the diffusion limit. Furthermore, they extend to various model rugged landscapes and to many experimental and experimentally motivated ones, including several experimental fitness landscapes involved in the evolution of antimicrobial resistance. This shows the robustness of our conclusions and their relevance to biologically relevant situations.

The time it takes to cross a fitness valley [59,60] and the entropy of trajectories on fitness landscapes [61] depend non-monotonically on *N*. However, both results arise from the possibility of observing double mutants in a wild-type population when *N* increases at fixed mutation rate *μ*. Small populations can also yield faster adaptation than larger ones [62,63], but this occurs at the onset of clonal interference. By contrast, we remained in the weak mutation regime, highlighting that even then, population size has non-trivial effects on adaptation. Our focus on weak mutation without strong selection (see also [20,21,27–29]) complements the study of strong selection with frequent mutation [33], going beyond the strong selection weak mutation regime.

The overshoot we find of the large-*N* limit of $\bar{h}$ is often small. In addition, it occurs for modest values of *N*, meaning that adaptation becomes most efficient for sizes that are quite small compared to the total size of many microbial populations. However, the relative amplitude of the overshoot, and the *N* at which it occurs, both increase with genome size *L* in *LK* landscapes. The large-*L* case is biologically relevant since genomes have many units (genes or nucleotides). Furthermore, in *LK* landscapes, the relative overshoot is largest for *K* = 1, i.e. pairwise epistasis, a case that describes well protein sequence data [48–50]. More generally, finite-size effects in early adaptation are expected for population sizes *N* such that *N*|*s*| is small for a sufficient fraction of mutations in the landscape, where *s* denotes the relative fitness effect of a mutation. Thus, finite-size effects should matter for larger population sizes if neutral and effectively neutral [22] mutations are abundant. This is a biologically relevant situation [51].

Besides, spatial structure and population bottlenecks yield smaller effective population sizes, for which our findings are relevant. Studying the effect of spatial structure on early adaptation in rugged fitness landscapes is an interesting topic for future work. Indeed, complex spatial structures with asymmetric updates or migrations impact the probabilities of fixation of mutations [64–68], which should affect early adaptation. Beyond the weak mutation regime, fitness valley crossing by tunnelling can aid adaptation [59,60], which may especially impact subdivided populations, as first discussed in Wright’s shifting balance theory [1,69] and shown in a minimal model [70]. Another interesting direction regards the effect of environment-induced modifications of fitness landscapes on adaptation [15,71].

## Data Availability

All relevant data are presented in the manuscript and electronic supplementary material [72]. Code is freely available at https://zenodo.org/record/7662530.
